# Absence of correlation between radiation-induced CD8 T-lymphocyte apoptosis and sequelae in patients with prostate cancer accidentally overexposed to radiation

**DOI:** 10.18632/oncotarget.26001

**Published:** 2018-08-24

**Authors:** Guillaume Vogin, Jean-Louis Merlin, Alexandra Rousseau, Didier Peiffert, Alexandre Harlé, Marie Husson, Labib El Hajj, Mihai Levitchi, Tabassome Simon, Jean-Marc Simon

**Affiliations:** ^1^ Institut de Cancérologie de Lorraine, Département de Radiothérapie, 54500 Vandœuvre-lès-Nancy, France; ^2^ UMR 7365 CNRS-Université de Lorraine, 54500 Vandœuvre-lès-Nancy, France; ^3^ Université de Lorraine, Faculté de Pharmacie, 54000 Nancy, France; ^4^ CNRS UMR 7039 CRAN Université de Lorraine, 54500 Vandœuvre-lès-Nancy, France; ^5^ Institut de Cancérologie de Lorraine, Service de Biopathologie, 54500 Vandœuvre-lès-Nancy, France; ^6^ APHP, Unité de Recherche Clinique de l'Est Parisien (URC-Est), Hôpital Saint Antoine, 75012 Paris, France; ^7^ APHP, Hôpital Universitaire de la Pitié Salpêtrière, Service de Radiothérapie, 75013 Paris, France

**Keywords:** late radiation-induced toxicity, radiation-induced lymphocyte apoptosis, prostate cancer, Epinal adverse event, individual radiation sensitivity

## Abstract

**Purpose:**

454 patients with prostate adenocarcinoma were accidentally overexposed to radiation in Epinal hospital, France, between August 1999 and January 2007. We aimed toevaluate whether radiation-induced CD4 or CD8 T-lymphocyte apoptosis (RILA) correlates with the severity of radiation toxicity.

**Methods:**

Between 2007 and 2013, all patients who received more than 108% of the prescribed radiation dose, after correction of the treatment plan, were convened, and blood was sampled at 6-months follow-up. Maximal Digestive toxicity (MDT) and maximal urinary toxicity (MUT) were graded using the Common Terminology Criteria for Adverse Events (NCI-CTCAE) v3.0 scale. RILA was assessed using flow cytometry.

**Results:**

245 patients were included in our study. After a median follow-up of 4.8 years, the MDT and MUT reached grade 3-4 in 37 patients and 56 patients, respectively. Patients with prostatectomy exhibited a statistically higher grade of MUT compared with those treated with definitive radiotherapy (*p*=0.03). The median RILA values were 11.8% and 15.3% for CD4 and CD8 T-lymphocytes, respectively. We found no significant correlation between CD4 or CD8 RILA and either MDT or MUT.

**Conclusion:**

RILA does not correlate with the inter-individual variation in MDT or MUT in the largest cohort of patients overexposed to radiation. The magnitude of the overdosage probably overrides biological predictors of toxicity, including individual radiosensitivity.

## INTRODUCTION

Inter-individual variation in the constitutional response of cells to radiation appears to play a major role in tissue homeostasis after radiation [1]. This so-called individual radiation sensitivity (IRS) has a Gaussian distribution in the population, with the most-radiosensitive 5% presenting the highest severity of late tissue reaction [2, 3]. Several biomarkers of IRS have been proposed as predictive assays of late toxicity such as radiation-induced CD8 T-lymphocyte apoptosis (RILA). Previous multicenter studies depicted a good negative predictive value in patients with high RILA values and low-grade late toxicity following conventional fractionated radiotherapy [4, 5].

Hypofractionation – accidental or not – increases the dose to which the tumor is exposed over a shorter period of time than standard treatment, with a related amount of healthy tissues theoretically overexposed to toxicity as reported in prostate cancer treatment [6]. Hypofractionated radiation could have specific radiobiological effects against tumor vasculature [7].

Approximately 3,000 patients worldwide have been reported to have experienced significant adverse events relating to radiotherapy (RT), with 38 patients (1.4%) reported to have died due to either radiation overdose toxicity or local control failure linked to an underdosage to the tumor [8–10].

**Figure 1:**
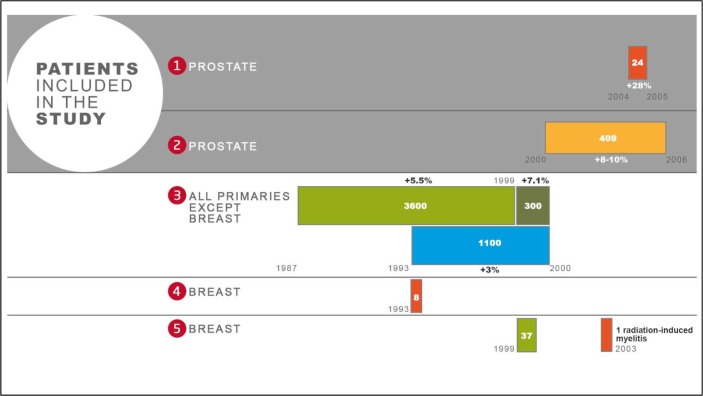
Flow chart of the Epinal adverse event Five successive and independent dysfunctions succeeded one another; the values in the rectangles indicate the number of patients affected by each event; the numbers in bold above or below the rectangles indicate the average value of the overdosage for each event.

Between 1987 and 2007, the Epinal adverse event involved the radiation overdosage of approximately 5,500 patients, the majority of whom suffered from late radiation-induced toxicity after treatment for prostate cancer [11]. This adverse event, which rated 6/7 on the French Society for Radiation Oncology (ASN/SFRO) scale, consisted of five episodes over the entire duration [12] (Figure 1)

- The most severe overexposure (firstly reported here) occurred in May 2004, following the introduction of dynamic wedge filters with manual entry of the number of monitor units (MU). Twenty-four patients treated for prostate cancer thereby received between 113% and 154% of the prescribed dose. This resulted in 8 patients presenting grade 3 or 4 (Common Terminology Criteria for Adverse Events; CTCAE v3.0) rectal complications, mostly pelvic necrosis and recto-vesical fistula, and the death of 13 patients, 10 following pelvic necrosis and one suicide. Other dysfunctions were disclosed *a posteriori* during the investigation.
- Between 2000 and 2007, 409 patients treated for prostate cancer suffered from overexposure related to portal imaging. These patients received between 108-110% of the prescribed dose. Of this group of patients, 66 developed grade 3 late radiation proctitis, and two died following a recto-vesical fistula.
- All patients treated for a non-breast primary cancer between 1987 and 2000 were over-irradiated following the setup of an isocentric 3D technique. The calculated dose was incorrect owing to misuse of homemade software, resulting in 5,012 patients receiving a relative overdose of 3–7.1% and the death of 2 patients.
- In 1993, 8 patients with breast cancer were overexposed following the implementation of a new device and dose calculation deviations with physical wedge filters.
- In 1999, 37 patients with breast cancer were over-irradiated owing to an inadequate radiation technique. Nine patients developed a severe late cardiac toxicity and one patient developed a late myelitis.

Interestingly, the severity of side effects observed in those patients significantly harmed in the Epinal adverse event was not necessarily proportional to the dose of radiation received. Therefore, we investigated the correlation between IRS and the severity of sequelae related to the magnitude of radiation overexposure.

## RESULTS

### RILA analyzed population

454 patients constituting the EPOPA cohort were followed between October 2008 and December 2013. Among them, 24 patients were greatly overexposed to radiation (i.e. approximately 120% of the prescribed dose). Of the cohort, 245 consented to blood sampling, five of whom received the aforementioned major overdose for a prescribed dose of 70-72 Gy (post prostatectomy radiation) and 74-78 Gy (definitive radiation). A total of 224 patient samples out of 245 (91.4%) were analyzed for RILA. The remaining 21 patient samples (8.6%) were excluded owing to: technical problems (N=12); uninterpretable results (N=7); or unavailability of samples for RILA (N=2). The 3 populations (RILA analyzed; non-analyzed; other EPOPA patients) were clinically comparable (Table 1). The mean time from the beginning of RT to blood sample collection was 5.1 ± 2 years.

**Table 1:** EPOPA patient characteristics

	RILA analyzed pts	Not analyzed included pts	Not included pts	Total EPOPA cohort
**Number of patients**	224	21	209	454
**Radical prostatectomy**	48 (21.4)^a^	5 (23.8)	30 (14.5)	83 (18.4)
**Concomitant hormone therapy**	64 (28.6)	1 (4.8)	44(21.3)	109(24.12)
**Comorbidities**	151 (67.4)	15 (71.4)	132 (65.0)	298 (65.5)
**Hypertension**	107 (70.9)	12 (80.0)	132 (73.7)	217 (72.6)
**Diabetes**	29 (12.9)	2 (9.5	20 (9.6)	51 (11.2)
**Smoking (active or ex-smoker)**	97 (43.3)	7 (33.3)	97 (46.4)	201 (44.3)
**Age at inclusion (years)**	73.8±6.2^b^	72.2±6.0	74.6±5.8	74.1±6
**Age at radiation therapy**	69.3±6.0	68.8±6.3	71.0±5.5	70.1±5.8
**Duration of radiation therapy (days)**	54.0±7.8	52.2±3.6	53.2±7.5	54.4±7.6
**Total dose received on the isocenter (Gy)**	78.3±4.3	78.5±7.7	79.1±5.8	78.7±5.2
**Total dose received on the isocenter per day (Gy)**	2.18±0.10	2.20±0.15	2.17±0.15^e^	2.18±0.13^e^
**Dose on the hottest 25% of rectum volume (Gy)^c^**	64.6±7.0	65.4±7.5	64.6±8.0	64.7±7.5
**Dose on the hottest 25% of bladder volume (Gy)^d^**	64.4±10.4	66.9±8.2	63.7±10.2	64.2±10.2

^a^ number of patients (%), ^b^ mean value ± standard deviation, ^c^ Total 61 missing values, 26 RILA analyzed patients, ^d^ Total 63 missing values, 27 RILA analyzed patients, ^e^: 2 missing values

The mean age at inclusion of the RILA analyzed population (N=224) was 73.8 years old, most of whom had preexisting co-morbidities (67.4%) including: hypertension (47.8%), diabetes (12.9%) and smoking (44.1%). Of this population, 22.8% received less than 75 Gy, 36.6% received between 75 and 80 Gy and 40.6% received 80 Gy or more. Irradiation after radical prostatectomy occurred in 48 patients with doses between 67.5 and 95.2 Gy (75.3 ± 4.2 Gy) (Supplementary figure 1). In addition, 42 patients received concurrent hormone therapy with a mean duration of 29.4 months (σ = 22.5 months). According to the cumulative dose-volume histograms (DVH), we reported D25%[Gy], i.e. the minimal dose delivered to the hottest 25% of the rectal or bladder wall [13, 14].

### Radiation-induced adverse effects

The whole EPOPA cohort and RILA-analyzed population showed similar maximal toxicity profiles (data not shown). In the RILA-analyzed population, after a median follow-up of 4.8 years [3.4-6.3], the maximal digestive toxicity (MDT) and maximal urinary toxicity (MUT) are reported in Table 2.

**Table 2:** Late digestive and urinary maximal toxicity profiles in the EPOPA patient population analyzed for RILA

	Nr of pts (%)
**Late digestive max. toxicity**^a^	
Gr 0-1	99 (44.2)
Gr 2	88 (39.3)
Gr 3-4	37 (16.5)
**Late urinary max. toxicity**^a^ pts with radical prostatectomy	
Gr 0-1	8 (16.7)
Gr 2	15 (31.2)
Gr 3-4	25 (52.1)
pts w/o radical prostatectomy	
Gr 0-1	54 (30.7)
Gr 2	66 (37.5)
Gr 3-4	56 (31.8)
total pts	
Gr 0-1	62 (27.68)
Gr 2	81 (36.16)
Gr 3-4	81 (36.16)

^a^ Digestive and urinary maximal toxicities were recorded according to the CTCAE v3.0 criteria

The majority of patients underwent grade ≥2 or higher maximal toxicity according to CTCAE v3.0 criteria (55.8% for digestive and 74.1% for urinary toxicity). Patients with prostatectomy exhibited a higher grade of urinary toxicity versus those with definitive RT (85.4 % *vs* 71.0%, *p*=0.03). In the most over-irradiated patients, we also observed other adverse effects including bladder and/or anal sphincter dysfunctions, and even tissue necrosis.

### RILA analyses

**Figure 2:**
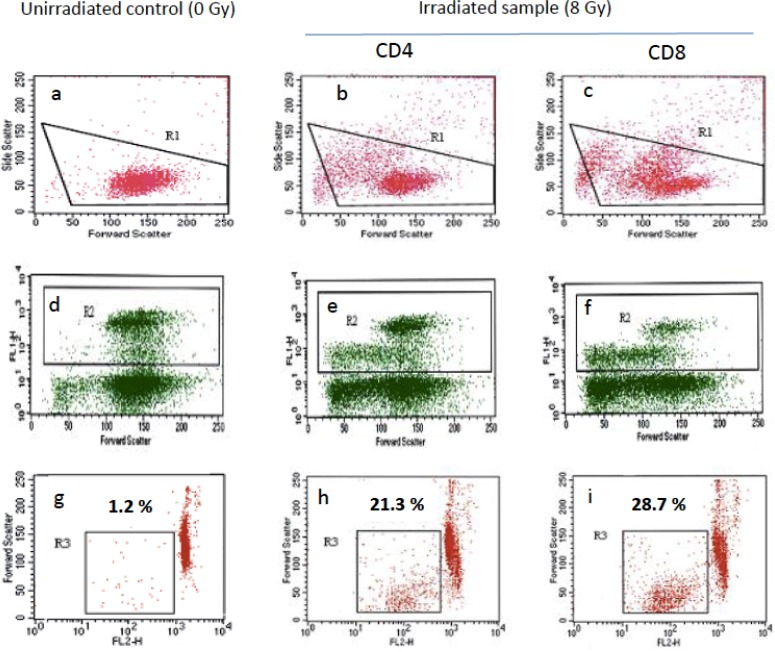
Radiation-induced lymphocyte apoptosis (RILA) flow cytometry profile Lymphocytes were selected (R1) from double-scatter dot plots **(a-c)** i.e. cell granularity (side scatter) and cell size (forward scatter). CD8 T-lymphocytes (R2) were then selected from R1 gated area as anti-CD4 or anti-CD8 FITC-conjugated antibody fixing cells (FL1H) **(d-f)**. CD4 (h) or CD8 (i) T-lymphocyte apoptosis rate (R3) was then calculated from the fraction of cells from R2 gated area with reduced size (forward scatter) and reduced DNA content i.e low propidium iodide labeling (FL2H). RILA was calculated after deduction of apoptosis rate of unirradiated controls **(g)** from irradiated samples **(h, i)**: respectively 20.1 and 27.5 % for CD4 and CD8 RILA in the present illustrative example.

**Figure 3:**
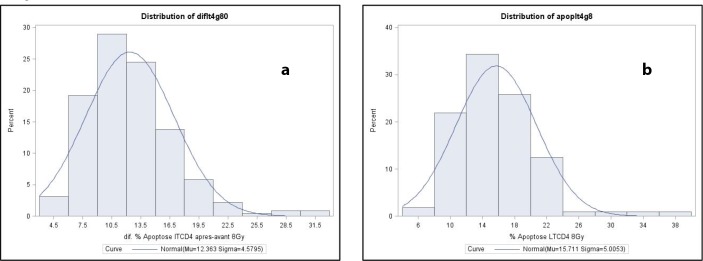
Distribution of 8 Gy-RILA in CD4 **(a)** and CD8 **(b)** lymphocytes in the EPOPA cohort patients.

RILA values were measured in 224 patients. Figure 2 illustrates a typical flow cytometry profile. Figure 3 shows the distribution of CD4 (Figure 3A) and CD8 (Figure 3B) T lymphocyte apoptosis. The RILA values exhibited a normal distribution with a median CD4 value of 11.8% (mean 12.4, SD 4.6, range 4.1–31.9) and a median CD8 value of 15.3% (mean 15.9, SD 5.8, range 4.4–37.7). First and second tercile cut-off values were calculated as 10.0% and 13.5% for CD4 RILA and 13.1% and 17.3% for CD8 RILA.

### RILA and cumulative incidence of digestive and urinary toxicities

A total of 118 digestive and 119 urinary grade ≥2 events occurred. Univariate analysis revealed a significant association between radical prostatectomy and a higher risk of grade ≥2 urinary toxicity (p=0.0158) and a trend for concurrent hormone therapy and the same endpoint (Table 3).

**Table 3:** Grade ≥2 digestive and urinary toxicity: Cox proportional hazard model - univariate analysis

Parameter		Digestive toxicity				Urinary toxicity			
		Hazard Ratio	95% Hazard Ratio Confidence Limits		p	Hazard Ratio	95% Hazard Ratio Confidence Limits		p
**Age at inclusion**		0.980	0.953	1.009	0.1724	0.981	0.954	1.010	0.1919
**Smoking**	Never	1			0.1216				0.2812
	Active or ex-smoker	0.743	0.511	1.082		0.817	0.565	1.181	
**Radical prostatectomy**	No	1			0.2378	1			0.0158
	Yes	1.284	0.848	1.943		1.627	1.096	2.415	
**Concurrent hormone therapy**	No	1			0.2807				0.0559
	Yes	1.237	0.840	1.821		1.444	0.991	2.105	
**Total dose received (Gy)**		1.007	0.964	1.053	0.7431	0.982	0.940	1.025	0.4090
**Total dose received (Gy)**	<75 Gy	1			0.7364	1			0.8162
	75 ≤ Dose <80 Gy	0.865	0.533	1.401		0.902	0.566	1.438	
	≥ 80 Gy	1.010	0.636	1.604		0.862	0.544	1.365	
**Dose on 25 % of rectum volume D25% (Gy)**		1.027	0.997	1.057	0.0759				
**Dose on 25 % of rectum volume D25% (Gy)**	<65	1			-				
	≥ 65	1.284	0.875	1.884					
	Missing	0.510	0.229	1.135					
**Dose on 25 % of bladder volume (Gy)**						1.002	0.984	1.020	0.8451
**Dose on 25 % of bladder volume (Gy)**	<75					1			-
	≥ 75					1.188	0.652	2.163	
	Missing					0.568	0.287	1.125	
**CD4 RILA %**		1.002	0.963	1.042	0.9290	1.033	0.997	1.071	0.0714
**CD4 RILA in tertiles**	CD4 RILA <10.0	1			0.8839	1			0.1542
	10.0≤CD4 RILA <13.5	0.935	0.598	1.462		1.384	0.870	2.202	
	CD4 RILA ≥13.5	1.045	0.674	1.620		1.559	0.988	2.460	
**CD8 RILA %**		1.004	0.974	1.035	0.8151	1.016	0.986	1.047	0.3046
**CD8 RILA in tertiles**	CD8 RILA <13.1	1			0.9363	1			0.5618
	13.1≤CD8 RILA <17.3	1.023	0.654	1.599		0.894	0.568	1.407	
	CD8 RILA ≥17.3	1.082	0.696	1.683		1.137	0.739	1.750	

- Type 3 tests are not valid

**Figure 4:**
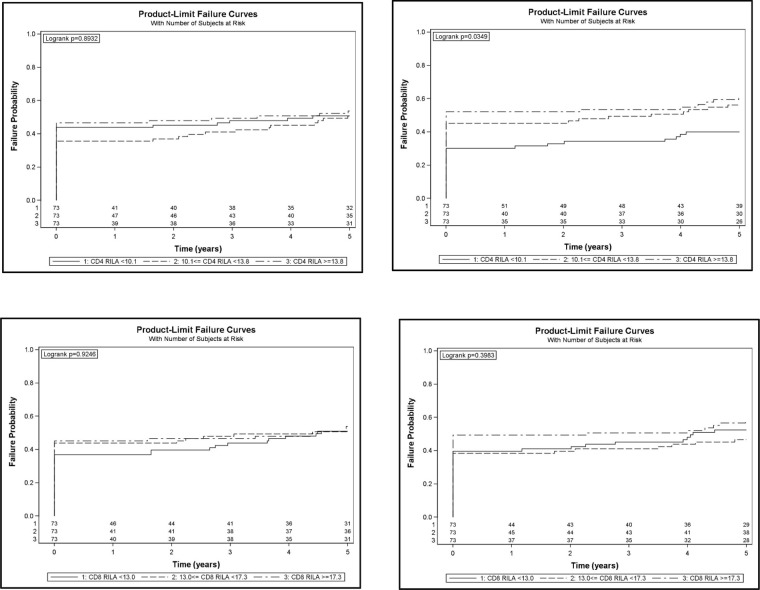
Cumulative incidence of grade ≥2 digestive (left panels) or urinary (right panels) toxicity according to CD4 (top panels) and CD8 (bottom panels) RILA

Our analysis revealed no significant correlation between CD4 and CD8 RILA and either MDT or MUT, even after adjustment (Supplementary Table 1). Patients who received a high radiation dose (≥ 80 Gy) and underwent grade 0-1 maximal toxicity (16.5% and 11.6% for MDT and MUT respectively) displayed similar CD4 and CD8 RILA levels to those measured in the other patients (Supplementary Table 2). There was however a significant correlation between CD4 RILA tercile and the cumulative incidence of grade ≥2 urinary toxicity (Figure 4). However, multivariate analysis failed to confirm this and revealed neither CD4 nor CD8 levels associated with the occurrence of grade ≥2 toxicity events (Supplementary Table 3).

## DISCUSSION

Predisposition to radiation-induced toxicity has primarily been explored by analyzing the IRS of normal tissue such as fibroblasts, skin biopsies and circulating lymphocytes [15]. Among these methods, the RILA assay showed an excellent negative predictive value for late radiation-induced toxicity after conventional dose fractionation in non-accidental situations in several tumor types, including prostate carcinomas, at several institutions [4, 5, 16].

Here, we used the FACS method originally developed, reported and validated by Oszahin and Azria [4]. The method was simplified during the two prospective French trials published after our analysis, applied only on CD8 cells showing the best correlation with late toxicity [5, 17]. In those studies, blood was collected prior the initiation of RT, and patients were followed during several years regarding toxicity. Other teams use annexin V – solely or associated with propidium iodide – to define the apoptotic subpopulation, with similar results [16, 18–21].

It should be noted that most the translational studies reported above were carried out after the completion of RT – patients who experienced toxicity were then selected for RILA measurement. In our study too, we assessed RILA after the occurrence of the accident and the constitution of the EPOPA cohort. Although the samples were collected after a median of 4.8 years [3.4–6.3] post RT, the radiation-induced apoptosis rate is a constitutional trait, the measurements of which are stable over time [22].

Our aim was to investigate whether RILA was linked to differences observed in the clinical outcome of these overexposed patients regarding late radiation-induced adverse effects. To our knowledge, no study has yet addressed this issue in such a clinical setting. Reactions of tissue and organs can vary with dose in both severity and frequency. When plotted on linear axes, a dose-dependent response is typically sigmoidal, with the effect becoming more frequent as the dose increases [23]. In the specific context of RT overdosage, the patients that were irradiated at the highest dose level and received unwanted hypofractionation – often exceeding the tolerated dose which causes a 5% risk of complications – were highly likely to suffer from tissue effects and with increased severity. Compared with routine RT practice, this high dosage could have a greater effect on resultant pathophysiology than any individual susceptibility, explaining our results.

However, hypofractionation is increasingly being used particularly in the treatment of prostate cancer [24]. Unlike the accidental context, safeguards are deployed to allow for a reduction of the volume of healthy tissues significantly irradiated through geometrical margins using, for example, precise, image-guided positioning and effective immobilization. Quality assurance is strengthened as a corollary.

IRS could thus have a lesser effect on late toxicity when radiation dosages are large. Two randomized trials evaluating the RILA test in non-accidental hypofractionation setting should provide answers.

Unlike our study, the original experiments defining the RILA assay [4] and the more recent study in patients with prostate cancer [25] were both performed using standardized prescribed doses.

Also, compared with populations included in previous studies our patients were exposed to confounding factors that affect RT tolerance such as the delivered dose over all, the advanced age of the patients along with a high co-morbidity rate, and the proportion of patients receiving concurrent hormone therapy, possibly further affecting the distribution of RILA values, as reported with letrozole [17] and tamoxifen [26] in breast cancer.

## MATERIALS AND METHODS

### Patient demographics

The overexposure of patients to radiation in the Epinal adverse event was discovered in March 2007. From April 2007, contact was made with patients who received the most significant overdose of RT (greater than 108% of the prescribed dose) treated in Jean MONNET hospital, Epinal, France between August 1999 and January 2007 for a prostate adenocarcinoma (events I & II on Figure 1) and were still alive. These patients constitute the “EPOPA” (Epinal Patients Overexposed for a Prostate Adenocarcinoma) cohort and were followed-up every six months. Toxicity was evaluated retrospectively from the first day of RT to the first follow-up visit, and prospectively from that day forward. To get metrics from the RT plans correlated with late toxicity, we retrospectively collected relevant DVHs on bladder and rectal walls once three-dimensional conformal RT had been implemented at the Epinal hospital (cohorts I & II only) [13, 14]. Blood was sampled from all consenting patients.

This study was approved by an ethical committee (Comité de Protection des Personnes) and the “Commission Nationale de l’Informatique et des Libertés” (CNIL) according to French regulations. The patients were fully informed and gave their consent. https://clinicaltrials.gov/ registered number: NCT00773656, retrospectively registered October 16, 2008.

### Grading of late radiation-induced adverse effects

Adverse effects were graded according to the NCI-CTCAE v3.0 toxicity scale [27].

### RILA assay

The RILA assay was performed as previously described using blood samples (5 ml) processed within 48hrs [4]. Briefly, 40 ml of total blood was collected in heparinized tubes. T-lymphocytes were isolated from whole blood by negative selection using rosette (RosetteSep®, StemCell Technology), followed by a Ficoll gradient (GE Healthcare). Lymphocytes were then cultivated in RPMI medium (Life technologies, France) with 10% FCS for 24 h at 37° C and 5% CO_2_. Half of the lymphocytes were irradiated *in vitro*. Irradiated and non-irradiated lymphocytes were then cultivated again at 37° C and 5% CO_2_ for 48 h. Samples received 8 Gy (6MV) with a dose debit of 1 Gy/min - corresponding to 1,600 MU (Clinac iX linear accelerator, Varian Medical Systems, France). RILA was analyzed using double-labeling flow cytometry (FACS Calibur, Becton Dickinson). CD4 and CD8 lymphocyte populations were selected using FITC-conjugated anti-CD4 (clone SK3) and anti-CD8 (clone SK1) mouse anti-human monoclonal antibodies, respectively (Becton Dickinson). The rates of CD4 and CD8 RILA were evaluated using PI (Sigma Aldrich, France) and sub-G1 peak calculation after acquisition of > 10,000 cell signals. RILA was defined as the population of cells with reduced DNA fluorescence and calculated as the percentage of total T-lymphocyte death induced by radiation dose (8 Gy) minus the spontaneous cell death (0 Gy). A radiosensitive patient is defined as a subject for whom the results of RILA assay are indicative of a low level of induced apoptosis, and preferably an induced apoptosis less than 16%. The intercenter reproducibility of RILA assay results has previously been validated [28].

### Statistics

CD4 and CD8 RILA were categorically coded according to tercile values. CTCAE MDT and MUT were recoded as grade 0-1 versus grade ≥2. Correlations between RILA tercile levels and quantitative variables were assessed using the Kruskall–Wallis test, whereas qualitative variables were assessed using either the Pearson chi-squared test or the Fisher exact test, where appropriate. A logistic regression model was used to assess the relationship between maximal toxicity and CD4/CD8 RILA independent of dose, radical prostatectomy, associated hormone therapy and smoking status (former or current smoker *vs.* never).

Censored data analysis: for each toxicity category, the time to grade ≥2 toxicity was defined as the time from the beginning of RT until the first occurrence of grade ≥2 toxicity. Follow-up was truncated at 5 years (median follow-up was 7.7 years).

The Kaplan–Meier estimaor (Greenwood variance) was used to assess cumulative incidence of grade ≥2 toxicity, and the log rank test was used to compare RILA tercile levels in univariate analysis. The Cox proportional hazard model was used to assess the association between RILA level and incidence of grade ≥2 toxicity independently of age at inclusion, smoking status, dose received on 25% of rectal or bladder wall volume, radical prostatectomy and associated hormone therapy. Analyzes were performed using SAS V.9.3 software (SAS Institute, Cary, North Carolina, USA).

## CONCLUSIONS

Overall, our results revealed no association between CD4 or CD8 RILA with either MDTs or MUTs in patients overexposed to radiation.

## SUPPLEMENTARY MATERIALS FIGURES AND TABLES



## Abbreviations

RT: Radiotherapy
CTCAE: Common Terminology Criteria for Adverse Events
IRS: Individual Radiation Sensitivity
EPOPA: Epinal Patients Overexposed for a Prostate Adenocarcinoma
RILA: Radiation-induced lymphocyte apoptosis
MUT: maximal urinary toxicity
MDT: maximal digestive toxicity.
