# Supplementary material for: Absence of correlation between radiation-induced CD8 T-lymphocyte apoptosis and sequelae in patients with prostate cancer accidentally overexposed to radiation
Source: Oncotarget. 2018 Aug 24;9(66):32680–9. doi: 10.18632/oncotarget.26001 (PMC6135683; doi:10.18632/oncotarget.26001)
Supplement: Supplementary file 1 [file oncotarget-09-32680-s001.pdf]

Absence of correlation between radiation-induced CD8 T-lymphocyte apoptosis and sequelae in patients with prostate cancer accidentally overexposed to radiation

SUPPLEMENTARY MATERIALS

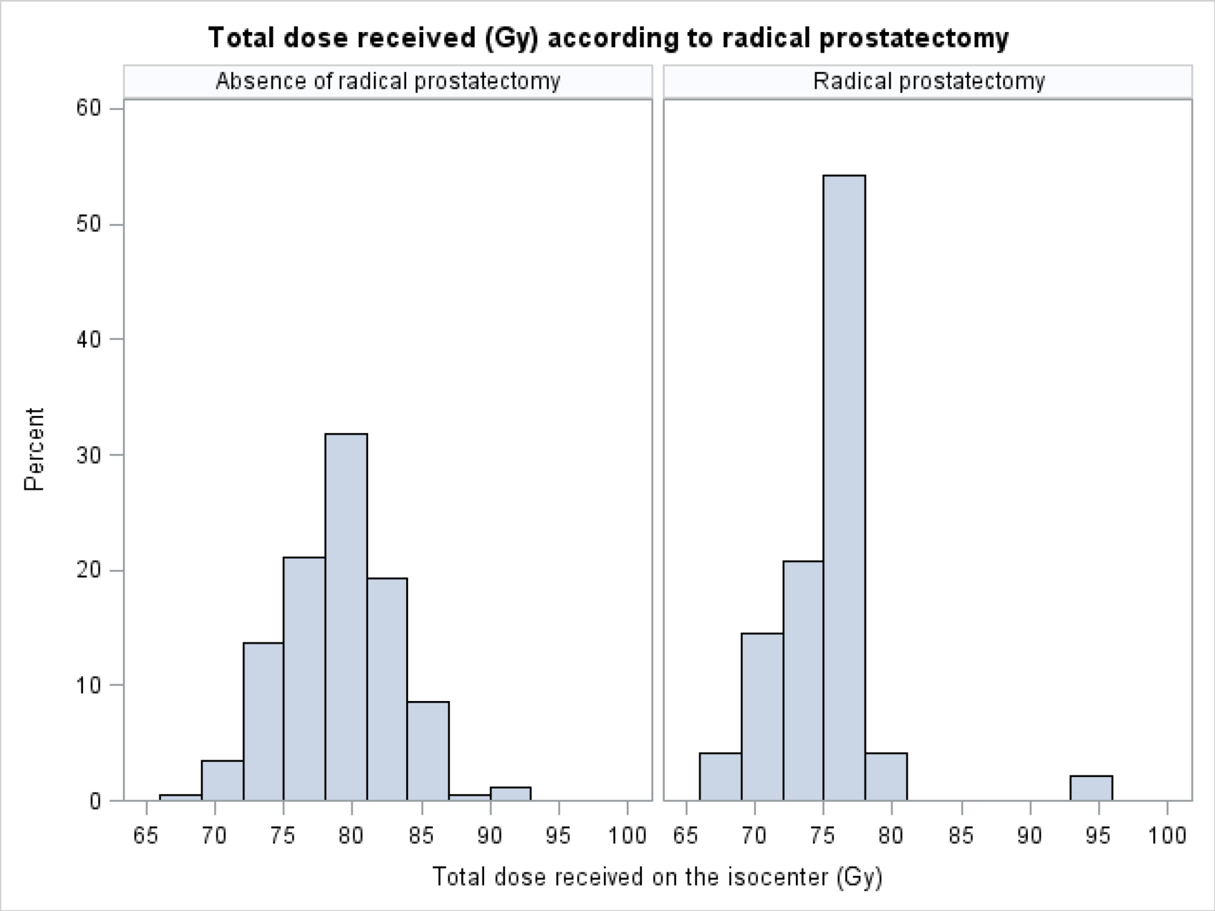

Supplementary Figure 1: Distribution of total dose in EPOPA cohort according to preliminary radical prostatectomy.

Supplementary Table 1: Maximal digestive (A, B) and urinary (C, D) toxicity and CD4 (A, C) and CD8 (B, D) RILA in EPOPA cohort in tercile distribution

		**Maximal digestive toxicity**		
**A**	**Gr 0-1**	**Gr 2**	**Gr 3-4**	**Total (%)**
**CD4 RILA <10.0**	31 (13.8)	30 (13.4)	13 (5.8)	74 (33.0)
**10.0≤CD4 RILA <13.5**	36 (16.1)	28 (12.5)	11 (4.9)	75 (33.5)
**CD4 RILA ≥13.5**	32 (14.3)	30 (13.4)	13 (5.8)	75 (33.5)
**Total (%)**	99 (44.2)	88 (39.3)	37 (16.5)	224 (100.0)
**B**	**Gr 0-1**	**Gr 2**	**Gr 3-4**	**Total (%)**
**CD8 RILA <13.1**	33 (14.7)	30 (13.4)	11 (4.9)	74 (33.0)
**13.1≤ CD8 RILA <17.3**	33 (14.7)	30 (13.4)	12 (5.4)	75 (33.5)
**CD8 RILA ≥17.3**	33 (14.7)	28 (12.5)	14 (6.3)	75 (33.5)
**Total (%)**	99 (44.2)	88 (39.3)	37 (16.5)	224 (100.0)
		**Maximal urinary toxicity**		
**C**	**Gr 0-1**	**Gr 2**	**Gr 3-4**	**Total (%)**
**CD4 RILA <10.0**	23(10.3)	26 (11.6)	25 (11.2)	74 (33.0)
**10.0≤CD4 RILA <13.5**	19 (8.5)	22(9.8)	34 (15.18)	75 (33.5)
**CD4 RILA ≥13.5**	20 (8.9)	33 (14.7)	22 (9.8)	75(33.5)
**Total (%)**	62 (27.7)	81 (36.1)	81 (36.2)	224 (100.0)
**D**	**Gr 0-1**	**Gr 2**	**Gr 3-4**	**Total (%)**
**CD8 RILA <13.1**	21(9.4)	25 (11.2)	28 (12.5)	74 (33.0)
**13.1≤ CD8 RILA <17.3**	19(8.5)	29(12.95)	27 (12.05)	75 (33.5)
**CD8 RILA ≥17.3**	22(9.8)	27(12.1)	26 (11.6)	75 (33.5)
**Total (%)**	62(27.68)	81(36.16)	81(36.16)	224 (100.0)

Supplementary Table 2: CD4 and CD8 RILA in patients exhibiting grade 0-1 digestive or urinary toxicity and having been exposed to more than 80 Gy in EPOPA

		N	CD4 RILA mean±SD	CD8 RILA mean±SD
**Digestive**	G0-1 and ≥ 80 Gy	187	12.3±4.7	15.9±5.9
	Others	37	12.5±4.1	15.9±5.4
**Urinary**	G0-1 and ≥ 80 Gy	198	12.4±4.7	16.0±5.9
	Others	26	12.2±3.7	15.1±5.1

Supplementary Table 3: Cumulative incidence of grade ≥2 digestive or urinary toxicity in EPOPA according to CD4 and CD8 RILA distribution

Digestive toxicity	2.5 years- cumulative incidence of grade ≥2 [IC95%]	5 years-cumulative incidence of grade ≥2 [IC95%]
**CD4 RILA <10.0**	47.3 [35.9-58.7]	52.8 [41.4-64.3]
**10.0≤CD4 RILA <13.5**	40.0 [28.9-51.1]	50.7 [39.4-62.0]
**CD4 RILA ≥13.5**	49.3 [38.0-60.6]	55.0 [43.6-66.3]
**CD8 RILA <13.1**	40.5 [29.3-51.7]	51.6 [40.2-63.1]
**13.1≤CD8 RILA <17.3**	48.0 [36.7-59.3]	52.0 [40.7-63.3]
**CD8 RILA >17.3**	48.0 [36.7-59.3]	55.1 [43.8-66.5]

Urinary toxicity	2.5 years- cumulative incidence of grade ≥2 [IC95%]	5 years-cumulative incidence of grade ≥2 [IC95%]
**CD4 RILA ≤10.0**	36.5 [25.5-47.4]	42.1 [30.8-53.4]
**10.0≤CD4 RILA <13.5**	48.0 [36.7-59.3]	56.0 [44.8-67.2]
**CD4 RILA ≥13.5**	54.7 [43.4-65.9]	62.0 [50.8-73.1]
**CD8 RILA ≤13.1**	44.6 [33.3-55.9]	50.1 [41.6-64.5]
**13.1≤CD8 RILA <17.3**	42.7 [31.5-53.9]	48.0 [36.7-59.3]
**CD8 RILA ≥17.3**	52.0 [40.7-63.3]	59.3 [48.0-70.5]
